# Identification of Geometrical Features of Cell Surface Responsible for Cancer Aggressiveness: Machine Learning Analysis of Atomic Force Microscopy Images of Human Colorectal Epithelial Cells

**DOI:** 10.3390/biomedicines11010191

**Published:** 2023-01-12

**Authors:** Mikhail Petrov, Igor Sokolov

**Affiliations:** 1 Department of Mechanical Engineering, Tufts University, Medford, MA 02155, USA; 2 Department of Physics, Tufts University, Medford, MA 02155, USA

**Keywords:** atomic force microscopy, cancer detection, machine learning methods

## Abstract

It has been recently demonstrated that atomic force microscopy (AFM) allows for the rather precise identification of malignancy in bladder and cervical cells. Furthermore, an example of human colorectal epithelial cells imaged in AFM Ringing mode has demonstrated the ability to distinguish cells with varying cancer aggressiveness with the help of machine learning (ML). The previously used ML methods analyzed the entire cell image. The problem with such an approach is the lack of information about which features of the cell surface are associated with a high degree of aggressiveness of the cells. Here we suggest a machine-learning approach to overcome this problem. Our approach identifies specific geometrical regions on the cell surface that are critical for classifying cells as highly or lowly aggressive. Such localization gives a path to colocalize the newly identified features with possible clustering of specific molecules identified via standard bio-fluorescence imaging. The biological interpretation of the obtained information is discussed.

## 1. Introduction

Cell phenotypes are traditionally defined by their genetic signature and the expression of specific genes and proteins [1]. There are multiple attempts to characterize cells using physical methods, such as various optical spectroscopy [2,3,4], cell mechanics [5,6,7], and electron microscopy analysis [8,9]. Recently, it has been demonstrated that atomic force microscopy can be used to image the cell surface with rather high precision and in a highly repeatable way [10,11,12]. Furthermore, Ringing mode of AFM imaging allows for the addition of up to eight additional maps of the physical properties of the cell surface, which are recorded simultaneously [13,14]. Combining just a few of these images, it was possible to separate human colorectal epithelial cells of two different aggressiveness with high accuracy [15]. This was done through classification analysis of the obtained data using machine learning (ML). However, it was impossible to identify particular surface features that are responsible for class identification because this information is hidden inside the ML algorithms. Furthermore, it is impossible to solve the inverse problem of restoring the cell surface using the developed ML algorithms. Thus, as of now, the physical and geometrical reasoning for the observed identification of cancer aggressiveness remains unknown.

Here, we suggest a ML approach to overcome this problem. To demonstrate our approach, we use the data recorded previously on human colon epithelial cancer cell lines of two different aggressiveness [15]. Specifically, we built maps of the probabilities of cells belonging to the highly aggressive type over the cell surface. Following the ideologically similar method of the convolutional neural network (CNN), we call these maps of probability “heatmaps”. It is instructive to note the differences between the class activation maps [16] used with deep convolutional neural networks (CNN) [17] and our approach. First, CNN requires quite a large number of data points to be trained. It is unrealistic to obtain with relatively slow AFM imaging. Secondly, the object we are trying to identify is unknown. This makes building CNN algorithms rather difficult. Furthermore, it was demonstrated that an efficient way to analyze the images is to convert the entire image into a set of surface parameters. This approach is completely different from the idea of the convolution of images. The approach described here deals with surface parameters. Essentially, the entire image is converted into a number in our approach. This is in contrast to another image in the CNN approach. Finally, the classification results mentioned above were obtained using regression and decision tree algorithms. To the best of our knowledge, the class activation maps were not built for non-CNN algorithms.

Here we demonstrate our approach using the Gaussian process regression algorithm. Instead of convolution, the decrease of the dimensional data space is achieved through the conversion of images into a set of surface parameters. Furthermore, the parameter set is further reduced by using the Gini importance index ranking and choosing only the top 10–20 most important parameters. The created data set is then randomly split into the testing and training subsets. The algorithm is created using the training subset. The created algorithm is then applied to a raster of images zoomed over the cell surface. The result is a map of the probabilities of cells belonging to the highly aggressive type over the cell surface. These regions are compared with the geometrical features of the height and adhesion images of the cell surface, which are simultaneously recorded. In other words, it allows us to identify the regions of the cell surface that are highly specific to high aggressiveness. We discuss the possible biological nature of the observed surface features responsible for the classification of cells as highly aggressive.

## 2. Methods

### 2.1. AFM Imaging of Cells Working in Ringing Mode

Although we used a pre-existing set of AFM images of cells [18], it is instructive to outline the advantages of Ringing mode modality [14,19] due to its novelty and because this imaging mode allows collecting maps of the physical properties of the samples rather than the surface topography. It can be used as an addition to sub-resonance tapping in AFM modes. In these modes, the AFM probe oscillates vertically in frequencies less than the resonance frequency of the AFM cantilever. On the lower approach to a sample, the AFM probe touches the sample. A typical AFM image is formed during such a touch. Ringing mode utilizes the part of the AFM probe trajectory right after the probe disconnects from the sample surface. Because the AFM probe typically develops some adhesion with the sample surface, the AFM cantilever jumps away from the sample surface after the probe disconnects from the sample. It results in free oscillations of the AFM cantilever. By analyzing these oscillations in real-time, it is possible to obtain information about the physical properties of the sample surface, such as its viscoelastic properties, the loss of energy during disconnection of the probe from the sample surface, and even the size of the molecules coating the sample surface. Presently, Ringing mode can provide 8–9 additional unique channels/maps of information. It is worth noting that all these images/maps are recorded simultaneously with regular scanning.

Here we analyze only three Ringing mode channels in addition to the regular height and adhesion channels: RM adhesion, RM restored adhesion, and RM viscoelastic adhesion. As was shown in Ref. [15], the regular height channel gave rather poor accuracy. The adhesion channel demonstrated many artifacts. Ringing mode channels provided the highest accuracy. Furthermore, when combining the height and the three Ringing mode channels together (the adhesion channel was dropped due to many artifacts), the accuracy of identification of the cell type reached 94% at the level of a single cell. In the present work, we demonstrate the proposed method using one of the Ringing mode channels, RM restored adhesion, as well as a combination of three Ringing mode channels and the height images.

### 2.2. Cells of the Study

The database of AFM images was assembled using cells from two human colon cancer cell lines, HT29 and Csk cells. [20,21] These two cell types are examples of colorectal cancer cells with two different degrees of neoplastic aggressiveness. Csk cells are shRNA-transfected HT29 cells; they demonstrate higher proliferation compared to the original HT29 cells. The cells were used in [15] to demonstrate the ability of AFM imaging combined with ML methods to identify each cell phenotype with rather high precision.

### 2.3. Machine Learning Method

The main idea of the proposed approach is to build a classifier trained and validated on the entire cell image/map and then apply this classifier to a zoomed-in area on the cell surface. We suggest using a regression classifier. For example, the applied classifier may give a probability of the cell belonging to the class of high aggressiveness. Moving each zoom over the cell surface, one can obtain a raster of the distribution of probabilities, which can be presented as a heatmap. The Gaussian process classifier is used in this work. Specifically, we used the algorithm of the Sklearn library. The RBF kernel was used [22]. The maximum number of iterations was chosen to be 1000.

**Figure 1:**
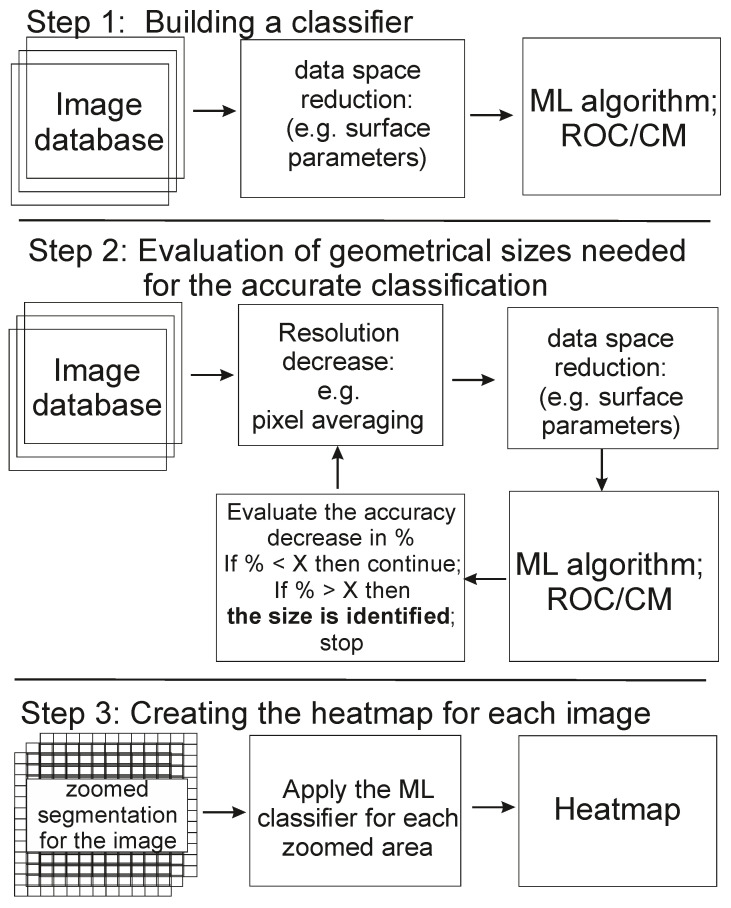
The flowchart of the proposed method. A suitable classifier is created in Step 1. The size of the zoomed area is defined in Step 2. The actual heatmaps are created in step 3 by applying the classifier algorithm developed in Step 1 to the zoomed-in areas of the size identified in step 2.

Figure 1 shows the workflow diagram of the described method. Step 1 implies the implementation of the method previously described in [15]. The goal of this step is to design a suitable ML algorithm to build a classifier to separate two classes of cells: low and high aggressiveness. The Gaussian process classifier is used in this work as an example. To avoid the problem of big data (the necessity of having a large database), the images are converted into a set of “surface parameters” [10,11,12]. These parameters are used in multiple engineering applications to characterize surfaces [23]. Examples of these parameters are as follows: roughness average, root mean square (RMS), surface skewness, surface kurtosis, etc. Definitions and abbreviations for these parameters are standard. Commercial software (SPIP by Image Metrology A/S, Denmark) was used to calculate all those parameters. This conversion substantially decreases the dimension of the data space. Instead of 512 × 512 pixels per image, each image is now characterized with ~40 surface parameters.

To decrease the dimension of the data space even further, the Gini importance index was used. All parameters were ranked by their importance for the classification. As was shown, ten parameters with the highest importance index were sufficient to achieve the high accuracy of classification per channel. When considering a combination of the three Ringing mode channels and height, it was sufficient to keep only the 20 parameters for the entire combination of the channels. It substantially accelerates the building and testing of the used classes.

Step 2 is needed to identify the size of the zoom area, which will be used for creating the heatmaps. Since there were no large physical structures previously found to identify the aggressiveness of cancer cells, we assume that such structures should be smaller than a particular threshold. To find this threshold, one can, for example, apply average filtering to decrease the effective resolution of the images. As the dimension of the filter kernel—the number of neighboring pixels used for averaging—increases, the corresponding images become more and more blurred, resulting in a lower classification accuracy.

Step 3 deals with the actual creation of the heatmaps. Each image is converted into a set of square zoomed areas of a fixed size identified in Step 2. The location of each zoom is shifted by one pixel (so the zoomed areas overlap) to move across the entire cell surface in a raster manner. The chosen surface parameters are calculated for the zoomed area. The calculated parameters are the input for the ML classifier developed in step 1. Application of the ML classifier to these parameters generates the probability of this zoomed area being classified as belonging to its class (e.g., the class of high aggressiveness for the cells of high aggressiveness).

## 3. Results

**Figure 2:**
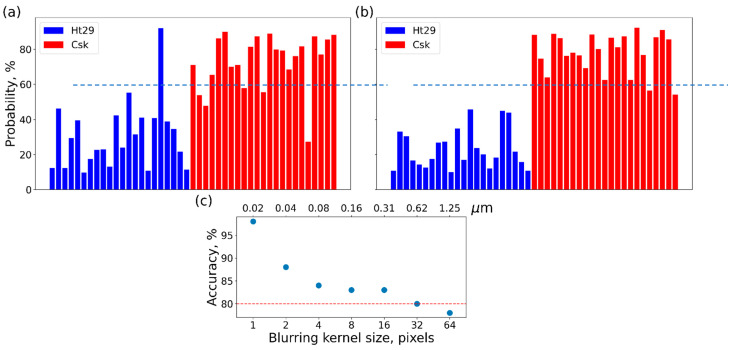
The results of Step 1: the regression classification of cells of two different aggressiveness when (**a**) using only one Ringing mode image (RM restored adhesion) or (**b**) using a combination of three Ringing mode images and the height of the image; (**c**) the decrease in the accuracy of classification (using the combination of channels) as a function of the pixel size of the averaging.

Figure 2 shows the application of the classifier to each individual cell. The probabilities of each cell belonging to the class of high aggressiveness are shown. Figure 2a shows an example of the analysis of the RM-restored adhesion channel. One can see that only four cells are misclassified when we choose 50% probability as a threshold for higher aggressiveness.

Figure 2b shows the results of that analysis of these combined channels. One can see that, on average, all cells are classified correctly if we choose 50% probability as a threshold for higher aggressiveness. These results are similar to what was reported in [15]. Figure 2c shows the results of Step 2 and the behavior of the classification accuracy for different sizes of the kernel used for blurring the images. Simple averaging is used here. For example, a size of 4 for the kernel means that each pixel of the original image is substituted with the value averaged over an area of 4 × 4 pixels. One can see that the accuracy starts to drop at a threshold of X = 80% if the size of the averaging size becomes greater than 32 × 32 pixels. Therefore, Step 3 will be executed using the zoom size of 32 × 32 pixels. It should be noted that the accuracy decreases rapidly, even with small averaging. We cannot, however, use those small numbers of pixels because we need a sufficient number of pixels to calculate the surface parameters. Therefore, we will keep the zoom size at 32 in this work.

**Figure 3:**
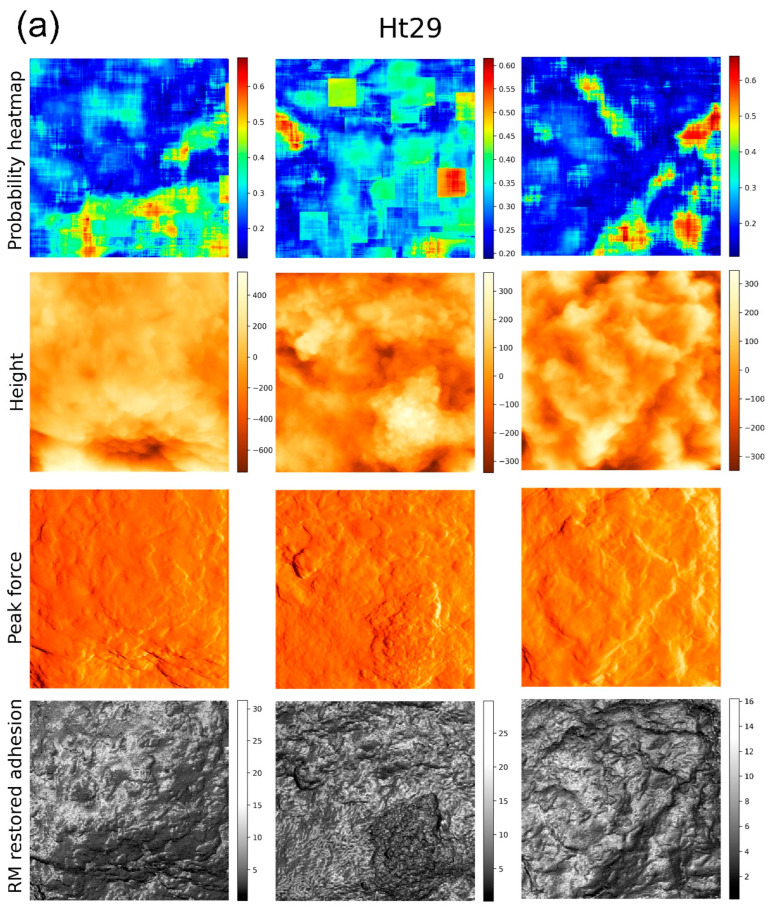

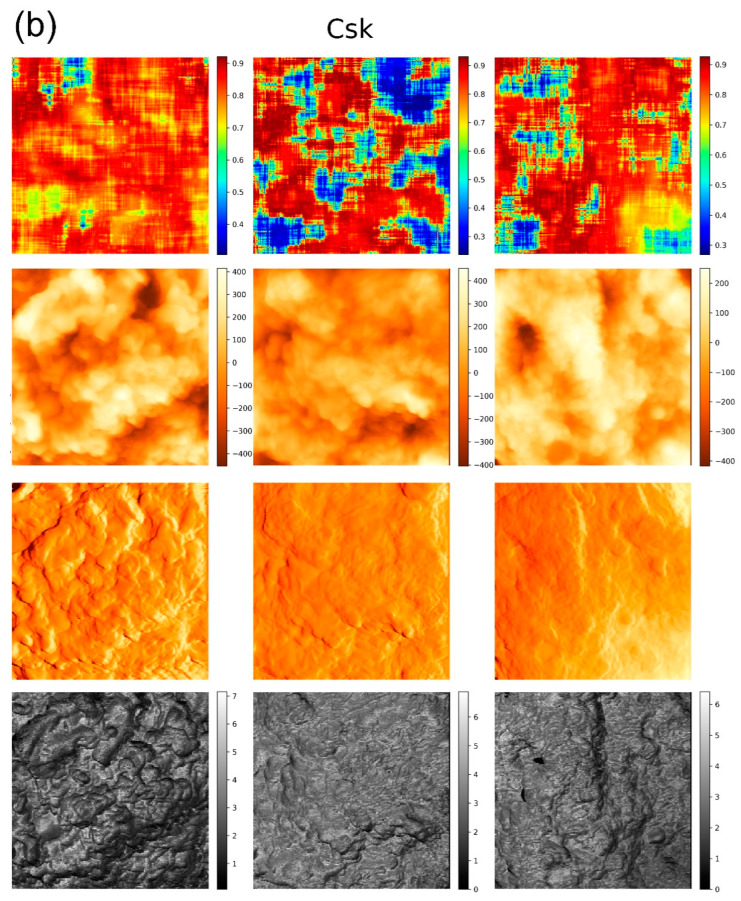
Heatmaps of cells of two different aggressiveness levels, low (**a**) and high (**b**), obtained when using one RM restored adhesion imaging channel. Three representative heatmaps for each class are shown. Each image represents 5 × 5 microns recorded with 256 × 256 pixels (a ¼ zoom of 10 × 10 microns recorded with 512 × 512 pixels). The top row shows the heat maps (probabilities for a 32 × 32 region around each pixel to belong to its class); the next row shows the height images of cells (the vertical scales are in nm); the third row shows the error of the feedback, PeakForce (in arbitrary units); and the bottom row shows RM-restored adhesion maps of the cells (the vertical scale is in nN).

Figure 3 demonstrates the results of the building of heatmaps for three representative cells of high aggressiveness and three heatmaps for the cells of lower aggressiveness. These heatmaps were obtained using only one RM-restored adhesion channel. One can clearly see the geometrical localization of the features responsible for higher aggressiveness. To correlate the obtained heatmaps with the geometrical features on the cell surface, the height as well as the PeakForce images are also shown. The PeakForce images are essentially the errors in the feedback. Such images are frequently presented because they are rather similar to photographs obtained with scattered optical microscopy or scanning electron microscopy. Here, these images are used to help identify the geometrical features specific to the regions of high versus low aggressiveness. Lastly, the actual imaging channel used for the classification, RM restored adhesion, is also shown beneath each cell.

**Figure 4:**
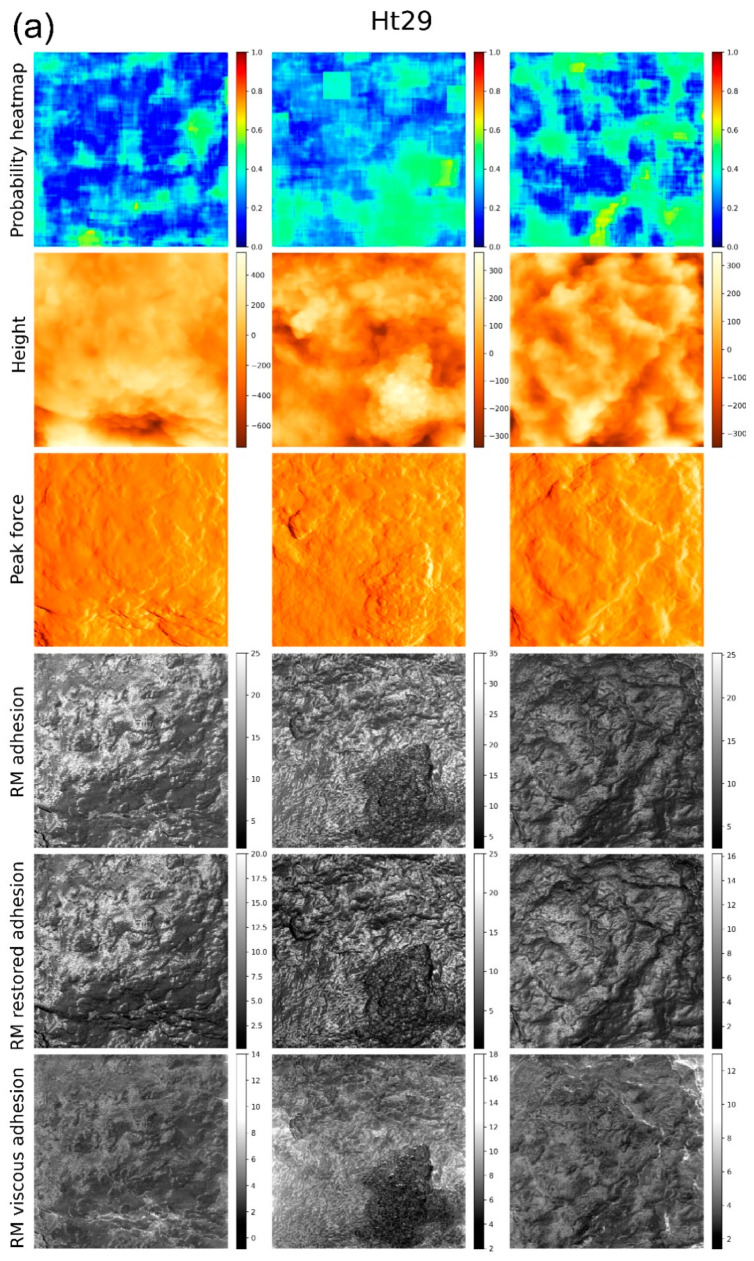

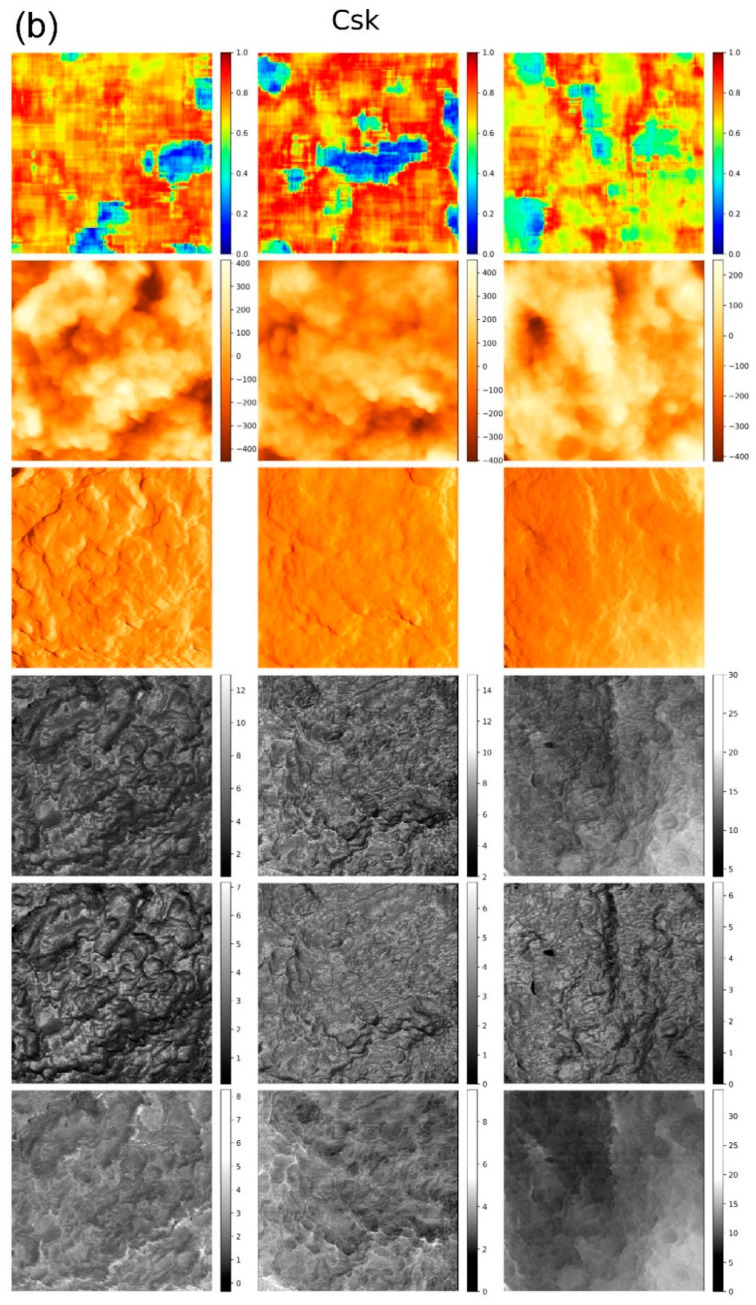
Heatmaps of cells of two different aggressiveness levels, low (**a**) and high (**b**), obtained when using a combination of the height channel and three RM imaging channels. Three representative heatmaps for each class are shown. Each image represents 5 × 5 microns recorded with 256 × 256 pixels (a ¼ zoom of 10 × 10 microns recorded with 512 × 512 pixels). The top row shows the heat maps (probabilities for a 32 × 32 region around each pixel to belong to its class); the next row shows the height images of cells (the vertical scales are in nm); the third row shows the error of the feedback, PeakForce (in arbitrary units); and the bottom three rows show RM adhesion, RM restored adhesion, and RM viscous adhesion maps of the cells (the vertical scales are in nN).

Figure 4 presents the heatmaps for the same cells obtained when the classifier is built using the combination of four different channels as we described above. Similar to the case of using just one Ringing mode channel, we show the heatmaps, the height, PeakForce, and three Ringing mode images that were used to build the classifier.

## 4. Discussion

**Figure 5:**
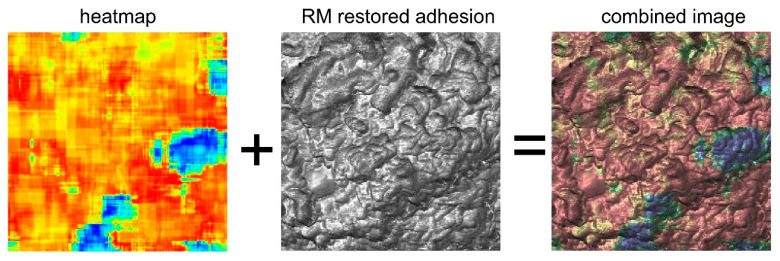
An example of a combination of heatmaps with one of the AFM images, RM restored adhesion. An example of a highly aggressive cell is shown.

First of all, the presented method effectively provides functional imaging of the cell surface. For example, the combination of heatmaps with one of the AFM images, RM restored adhesion, is shown in Figure 5. One can see a clear localization of areas with low aggressiveness on the RM restored adhesion image. Such localization gives a path to colocalize the newly identified features with possible clustering of specific molecules by using standard biological fluorescence imaging. There are plenty of studies of fluorescent imaging of malignant cells by using fluorescent markers specific to proteins and receptors associated with the progression toward cancer. While it is interesting to look for a possible colocalization, it may well be that the described method shows completely different information from what can be obtained with just fluorescent labeling. After all, we analyze the distribution of physical properties over the cell surface. Such properties are presumably created by multiple combinations of various molecules and biochemical pathways. From this point of view, the molecular origin of this property can be essentially nonlocal. It is highly probable that the present method can be complementary to the existing biological approaches and is expected to be synergistic with the known biological characteristics of cells.

In the present work, we also try to demonstrate the possibility of identifying specific features on the cell surface that are responsible for the classification of cells as higghly or less aggressive. The first clue about it can be seen in Figure 2c. One can see that the geometrical features of the order of 20–40 nm are rather important for the classification. When these features are averaged out, the accuracy drops substantially. The original images of cells were recorded with 512 × 512 pixels for an area of 10 × 10 µm^2^. So each pixel is approximately 20 nm. Going with the averaging further, one can see a relative plateau down to the averaging size between 16 and 32 pixels, which corresponds to ~0.5 microns. When we average out the surface features above this size, the accuracy drops further. Thus, we can speculate that geometrical features of ~0.5 microns are the second-most important contributors to the classifier.

**Figure 6:**
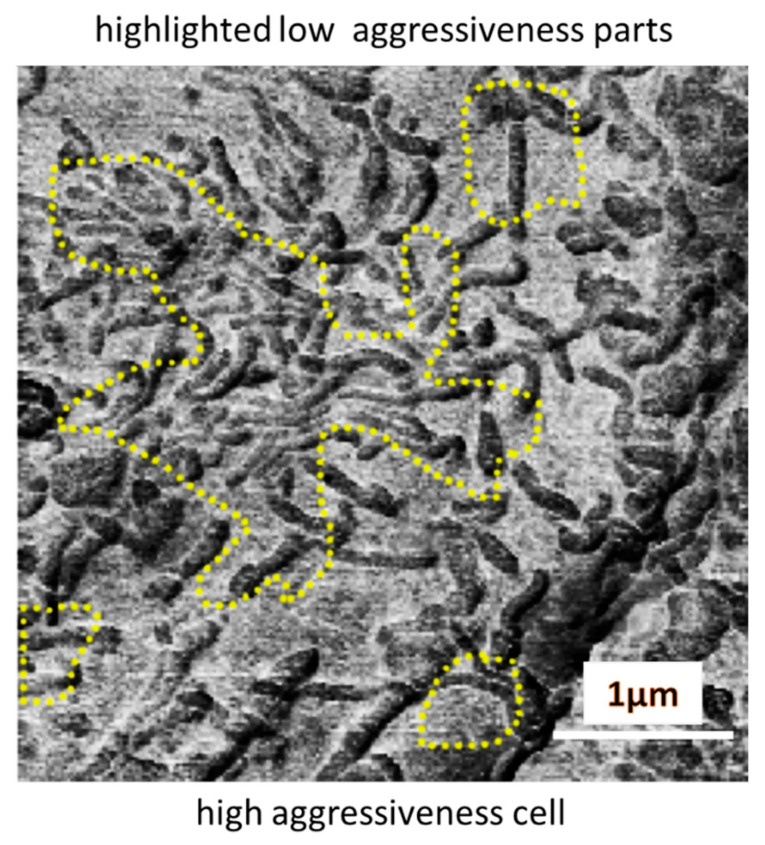
The regions of low aggressiveness are shown on a highly aggressiveness cell. The RM-restored adhesion image is shown.

Can we identify the type of biological features based on the above size information? Identification of biological features of the order of 20 nm can be pretty challenging because it is the size of a single antibody. For example, we may deal with the distribution of particular receptors on the cell surface. As to the size of the surface features of ~0.5 microns, these are presumably microvilli and microridges, corrugations of the cellular membrane created by F-actin fibers surrounded by the pericellular membrane. Although the diameter of these fibers can be of the order of 10 nm, the length of these fibers is within the submicron range. Figure 6 shows an example of a highly aggressive cell surface (the RM-restored adhesion is shown) with highlighted regions that were identified by the ML algorithm as the parts of low aggressiveness. The microvilli are clearly seen on the cell surface. One can speculate that the largest region of low aggressiveness is associated with a higher density of microvilli. Presumably, such a “one-dimensional” attempt to identify areas specific to high and low aggressiveness is not accurate. While it is an interesting way of thinking about high versus low aggressiveness, the microvilli density is definitely not the only indicator needed for precise identification. Even in the same image, one can see the (smaller) regions that do not show high microvilli density.

**Figure 7:**
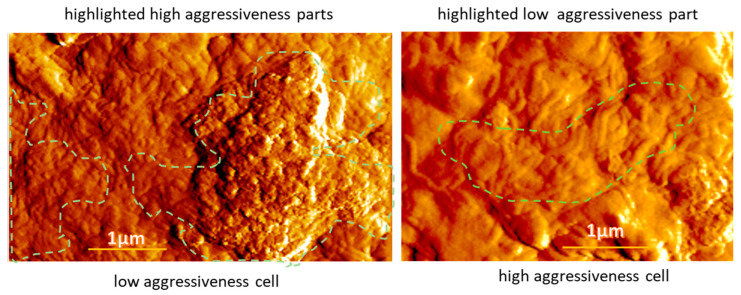
Localization of areas of different aggressiveness on cells of low and high aggressiveness (outlined by dashed lines). (**Left**): Areas of high aggressiveness are shown on the cell image of a low aggressiveness cell. (**Right**): An area of low aggressiveness is shown on the cell image of a high-aggressiveness cell. The PeakForce AFM images are shown.

Figure 7 shows examples of the difficulty of using one particular parameter to separate high and low aggressiveness. Zoomed-in areas of cells with low and high aggressiveness are shown. In these images, there are areas of specific high and low aggressiveness identified by the heatmaps. The PeakForce AFM images are shown in this case. These images provide a good highlight of the small surface features of the samples. One can see the areas of small features sized between 20–40 nm. Such areas are clearly seen on the low-aggressive cells (left image of Figure 7). Compared to the high aggressiveness of that cell, one might conclude that the higher clustering of these creatures is an indication of high aggressiveness. However, compared to the high aggressiveness cell image (the right image of Figure 7), one can see almost no such features. Interestingly, the low aggressiveness part seems to be associated with microvilli clustered in the middle of the image, which is similar to the case shown in Figure 6. Based on these examples, one can conclude that focusing on just one surface feature does not work to explain the observed heatmaps.

There is one more strong argument in favor of the above statement. One can compare the locations of the regions of high and low aggressiveness shown in Figure 3 and Figure 4. These regions were found on the same cells using either just one imaging channel (Figure 3) or a combination of four imaging channels (Figure 4). One can see that these regions are not necessarily the same on the same cells. Taking into account that the combination of four different channels gives much higher accuracy, we can conclude that just the surface geometry is unlikely to be sufficient to identify cell aggressiveness with sufficiently high precision. To attain high precision, one needs to combine geometrical features with the physical properties of the cell surface. We expect that the described approach will allow identifying a connection between the physical and biochemical properties of the cell surface by colocalization of functional biochemical images and the images shown, e.g., in Figure 5. Further, high-resolution imaging will be helpful for more precise localization of the surface features. We plan to do that in future work.

In conclusion, it is worth noting that the present method is described in an example cell line. It is justified by the fact all cells within each line are presumably the same. Expanding this method to a particular clinical task would require the study of a large number of samples due to the variability of cells within each tumor and the diversity of patients. This is another task for future work.

## Data Availability

The data presented in this study are available on request from the corresponding author.
